# Molecular Genetic Analysis and Prenatal Diagnosis of a Family With *MECP2* Duplication Syndrome

**DOI:** 10.1002/jcla.70373

**Published:** 2026-10-15

**Authors:** FengQiu Lin, DingGuo Chen, ZhengLin Lu, Yu Huang, ChunFang Wang, JuHua Liang, XueJuan Nong, ZhengYi Chang, FuBo Wei, GuiDan Xu

**Affiliations:** ^1^ Department of Laboratory Center Affiliated Hospital of Youjiang Medical University for Nationalities Baise Guangxi Zhuang Autonomous Region China; ^2^ Graduate School of Youjiang Medical University for Nationalities Baise Guangxi Zhuang Autonomous Region China; ^3^ Key Laboratory of Research on Clinical Molecular Diagnosis for High Incidence Diseases in Western Guangxi of Guangxi Higher Education Institutions, Education Department of Guangxi Zhuang Autonomous Region Baise Guangxi Zhuang Autonomous Region China; ^4^ Guangxi Engineering Research Center for Precise Genetic Testing of Long‐dwelling Nationalities Baise Guangxi Zhuang Autonomous Region China

**Keywords:** genetic analysis, high‐throughput sequencing, *MECP2* duplication syndrome, multiplex ligation‐dependent probe amplification, prenatal diagnosis

## Abstract

**Objective:**

This study aimed to conduct a molecular genetic analysis and prenatal diagnosis for a family with *MECP2* duplication syndrome (MDS), with the goal of improving diagnostic accuracy and enabling informed reproductive decisions to reduce the incidence of this condition.

**Methods:**

The study involved a proband (a 15‐week‐old fetus), his symptomatic older brother, and their parents. Genomic DNA from chorionic villus and peripheral blood samples was analyzed using high‐throughput sequencing to detect chromosomal copy number variations (CNVs). To confirm the findings, multiplex ligation‐dependent probe amplification (MLPA) was specifically employed.

**Results:**

High‐throughput sequencing identified a 0.35 Mb duplication at Xq28 in the fetus and his mother, and a 0.4 Mb duplication in the brother. The duplicated region contained the critical *MECP2* gene. MLPA analysis confirmed the *MECP2* gene duplication in the fetus, leading to a definitive diagnosis of MDS. The mother was identified as an asymptomatic carrier.

**Conclusion:**

Both the proband and his brother were diagnosed with MDS, having inherited the duplication from their asymptomatic mother. The combined use of high‐throughput sequencing and MLPA proved to be an effective strategy for accurate pedigree analysis and prenatal diagnosis of MDS, providing a crucial basis for genetic counseling.

## Graphical abstract

Combined low‐pass CNV‐seq and MLPA identified an inherited Xq28 duplication encompassing *MECP2* in a 15‐week male fetus and his affected older brother, transmitted from an asymptomatic mother. This integrated strategy enabled accurate pedigree analysis and prenatal diagnosis of *MECP2* duplication syndrome, supporting genetic counseling and reproductive decision‐making.

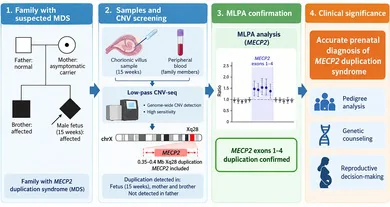

## 1 Introduction

*MECP2* duplication syndrome (MDS) comprises a spectrum of clinical manifestations caused by duplication of the *MECP2* gene located in the Xq28 region of the X chromosome. It is an X‐linked recessive neurodevelopmental disorder and accounts for approximately 1% of cases of X‐linked intellectual disability [1]. Most affected individuals are male, with an estimated live‐birth incidence of approximately 1 in 150,000. Approximately 50% of patients die before 25 years of age, primarily because of severe infections and recurrent epileptic seizures [2, 3]. The *MECP2* gene encodes methyl‐CpG‐binding protein 2, a critical epigenetic regulator in neurodevelopment that plays an essential role in neuronal maturation, synapse formation, and gene expression regulation. Duplication of this gene results in overexpression of the *MECP2* protein, with expression levels approximately 2‐ to 6‐fold higher than normal, thereby disrupting normal neural network function and leading to intellectual disability, hypotonia, and other related symptoms. MDS follows a typical X‐linked recessive inheritance pattern, with affected males showing complete penetrance and severe phenotypes. It accounts for approximately 2% of cases in males with intellectual disability accompanied by severe progressive neurological symptoms [4]. Female carriers are often asymptomatic because of skewed X‐chromosome inactivation, but they can transmit the pathogenic variant to their offspring [5]. Carrier mothers have a 50% probability of transmitting the mutation to their sons, who will be affected, and a 50% probability of transmitting it to their daughters, who will become carriers. Given the lack of effective treatment for this disorder, pedigree analysis and prenatal diagnosis have become essential strategies for preventing the birth of affected children. As MDS is a rare disease with limited clinical reports, this study aimed to perform molecular genetic analysis and prenatal diagnosis in a family affected by MDS in order to improve early diagnostic capability, enhance disease management, and optimize clinical outcomes.

## 2 Subjects and Methods

### 2.1 Subjects

The proband was a 15‐week‐old fetus evaluated at Affiliated Hospital of Youjiang Medical University for Nationalities. The proband's mother was 37 years old, representing advanced maternal age, and had a history of adverse pregnancy outcomes. She had a normal phenotype, no history of consanguinity, and one living child. The proband's older brother presented with severe intellectual disability, emotional instability, typical autistic behaviors, motor developmental delay, abnormal muscle tone, limb rigidity, occasional epileptic seizures, and immunodeficiency. Based on the mother's adverse obstetric history and the clinical manifestations observed in the older sibling, the fetus underwent combined high‐throughput sequencing and multiplex ligation‐dependent probe amplification (MLPA). In addition, peripheral blood samples were collected from the proband's parents and older brother for high‐throughput sequencing to confirm the diagnosis and perform pedigree analysis.

### 2.2 Methods

#### 2.2.1 Genomic DNA Extraction

After written informed consent was obtained, genomic DNA was extracted from the fetus and family members using a genomic DNA extraction kit (Guangzhou Darui Biotechnology Co. Ltd.) according to the manufacturer's instructions. DNA purity and concentration were measured using a Qubit 2.0 Fluorometer and the dsDNA HS Assay Kit. The extracted DNA samples were stored at −20°C until further analysis.

#### 2.2.2 High‐Throughput Sequencing for Chromosomal Copy Number Variation (CNV) Analysis

Constructed DNA libraries were sequenced using the DA8600 high‐throughput sequencer (Guangzhou Darui Biotechnology Co. Ltd.) with an average sequencing depth of 0.2× and coverage of ≥ 99%. The sequencing data were analyzed bioinformatically by aligning each read to the reference genome (GRCh37/hg19), followed by standardized *Z*‐score analysis to detect chromosomal abnormalities. A *Z*‐score threshold of ±3 was used to define significant copy number changes. Public databases, including DECIPHER, UCSC, OMIM, ClinGen, ClinVar, DGV, and PubMed, were consulted to assess the clinical significance of the detected CNVs. In the present study, the 0.2× CNV‐seq result was used primarily for CNV screening and family segregation analysis rather than definitive breakpoint resolution. The fetal *MECP2* duplication was independently confirmed by MLPA. Interpretation of CNVs was performed in accordance with the American College of Medical Genetics and Genomics (ACMG) guidelines for CNVs.

#### 2.2.3 MLPA Testing and Result Analysis

MLPA analysis was performed using the SALSA MLPA Probemix P015 *MECP2* kit (MRC‐Holland, Amsterdam, Netherlands) according to the manufacturer's standard protocol. This probe mix contains 24 probes targeting the X chromosome, including four probes covering exons 1–4 of the *MECP2* gene, and eight reference probes targeting autosomal genes (including *TUBB3*, *GAPDH*, and others). Raw data were analyzed using Coffalyser. Net software. The signal intensity of each target probe was normalized to the average signal intensity of the reference probes. The copy number cut‐off values were defined as follows: < 0.7 indicated a deletion, 0.7–1.3 indicated a normal copy number, and > 1.3 indicated a duplication.

## 3 Results

### 3.1 Whole‐Genome CNV Analysis by High‐Throughput Sequencing

High‐throughput sequencing of chorionic villus samples from the pregnant woman revealed a 0.35 Mb duplication at chrX: 153,000,000–153,350,050 (GRCh37/hg19), indicating the presence of an Xq28 duplication on the fetal X chromosome (Figure 1A and Table 1). The identified duplicated fragment showed substantial overlap with a ClinGen dosage‐sensitive region with a triplosensitivity score of three. This region corresponds to Xq28, encompassing *MECP2*, located at chrX:153,273,980–153,375,749 in GRCh37/hg19 (Figure 2). Given the ultra‐low sequencing depth (0.2×), the reported CNV boundaries and size should be regarded as approximate rather than definitive. The reported duplicated region involved 15 protein‐coding genes/loci, including *MECP2* (*OMIM300005*), *IRAK1* (*OMIM300283*), *L1CAM*, *NAA10*, *AVPR2*, *ABCD1*, *SSR4*, and *HCFC1*. Peripheral blood analysis of the mother revealed an approximately 0.35 Mb pathogenic duplication in the same Xq28 region, consistent with the chorionic villus results, suggesting maternal inheritance of the fetal duplication (Figure 1B and Table 1). Peripheral blood analysis of the proband's brother also identified a pathogenic 0.4 Mb duplication at chrX:152,949,977–153,350,520 (GRCh37/hg19) (Figure 1C and Table 1). No pathogenic CNV was detected in the father (Figure 1D and Table 1). Representative previously reported cases of MDS from China and other countries are summarized in Table 2 for comparison with the present family [6, 7, 8, 9, 10, 11, 12, 13].

**FIGURE 1:**
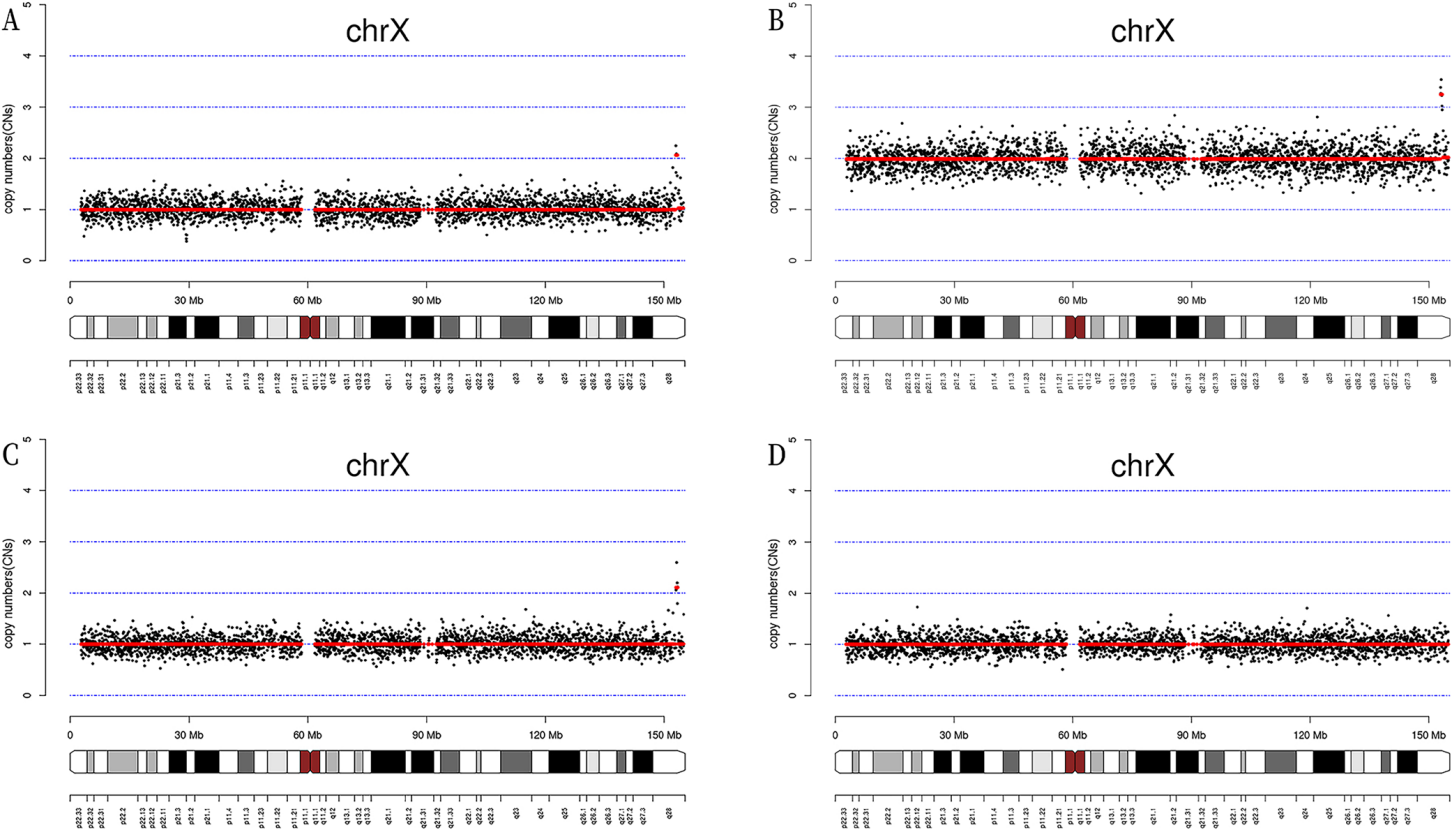
High‐throughput sequencing results. Panels A–D show the CNV‐seq results for the fetus, mother, brother, and father, respectively.

**TABLE 1:** High‐throughput sequencing results of the family members (GRCh37/hg19).

	Chromosomal region	Genomic location [hg19]	Copy number status	Segment length
Fetus	Xq28	chrX:153,000,000–153,350,050	duplication	0.35 Mb
Fetal mother	Xq28	chrX:153,000,000–153,349,885	duplication	0.35 Mb
Fetal brother	Xq28	chrX:152,949,977–153,350,520	duplication	0.4 Mb
Fetal father	No abnormality detected	—	—	—

**FIGURE 2:**
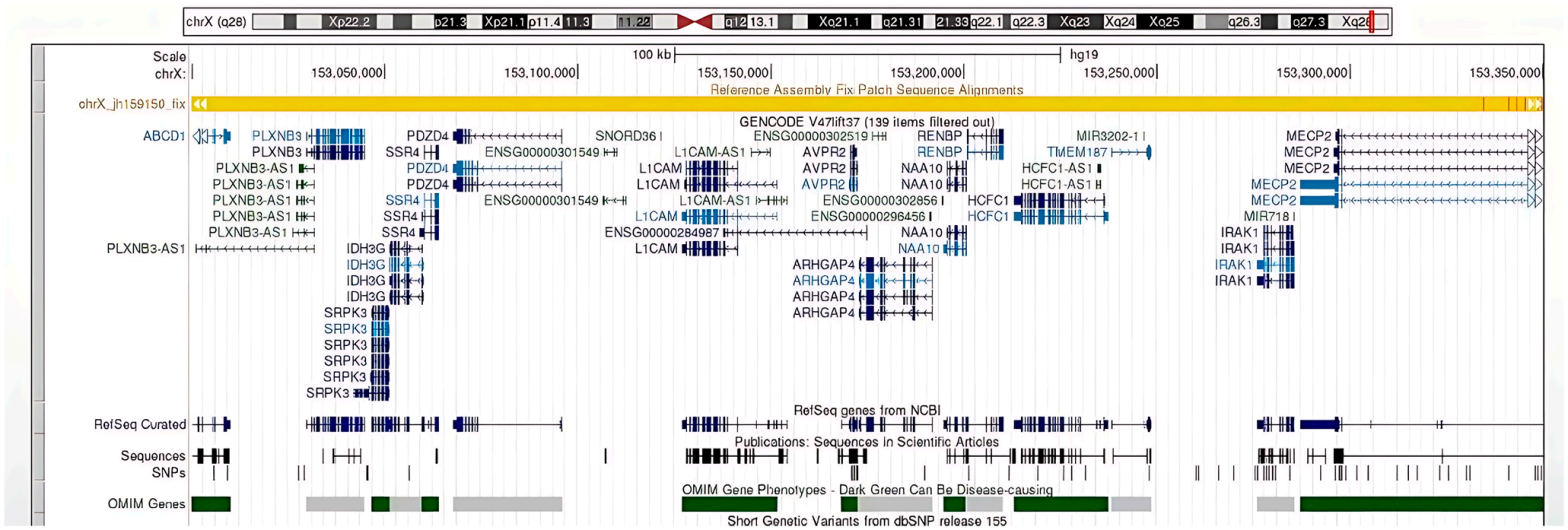
Schematic diagram of the duplicated Xq28 fragment detected in the fetus by high‐throughput sequencing. The figure shows the location, size, and genes involved in the duplicated region.

**TABLE 2:** Representative cases of MDS reported in China and other countries in recent years.

References	Gender	Age	Inherited/de novo	Duplication size[hg19]	Phenotype
Kim et al. [6]	Male	30 years	Inherited	Xq28 duplication breakpoint located at 153,297,646–153,298,018 bp	Intellectual and speech impairment, epilepsy, progressive spasticity, etc.
Zeng et al. [7]	Male	2 years	Inherited	Xq28 (140,360,001–154,810,001), 14.45 Mb	Developmental delay, recurrent infections, epilepsy, gastrointestinal issues, etc.
Zeng et al. [7]	Female	23 years	Inherited	Xq28 (139,460,001–154,810,000), 15.35 Mb	Midfacial retrusion
Gongang et al. [8]	Male	8 years	Inherited	Xq28 (152,780,136–153,390,314), 610 kb	Recurrent infections, global developmental delay with aphasia, epilepsy, craniofacial dysmorphisms, etc.
Yon et al. [9]	Male	10 years	Inherited	Xq28 (153,027,303–153,438,781), 411 kb	Global developmental delay and intellectual disability, autism, facial dysmorphisms, epilepsy, etc
Abdala et al. [10]	Male	25 years	De novo	Xq28 (153,263,517–155,260,560), 2 Mb	Developmental delay, intellectual disability, autism, recurrent infections, facial dysmorphisms, etc.
Chow et al. [11]	Male	3 years	Inherited	Xq28 (152,257,222–153,158,798), 4.96 Mb	Developmental delay, intellectual disability, epilepsy, recurrent infections, constipation, etc.
Ha et al. [12]	Male	8 years	Inherited	Xq28 (153,101,077–153,609,163), 508 kb	Developmental delay, hypotonia, speech delay, cognitive and motor impairment, etc.
Ha et al. [12]	Male	12 years	Inherited	Xq28 (152,930,233–153,230,284), 0.091 kb	Developmental delay, intellectual disability, epilepsy, recurrent infections, etc.
Pascual‐Alonso et al. [13]	Female	6 years	De novo	Xq28 (153,194,797–153,541,289), 0.346 kb	Intellectual disability, delayed speech development, stereotypic behaviors, impaired pain perception, etc.

*Note:* All genomic coordinates are based on the GRCh37/hg19 reference genome.

### 3.2 MLPA Results

MLPA analysis using the P015 probe mix confirmed that the signal intensities of the *MECP2* gene probes in the fetal Xq28 region were all > 1.3 (range, 1.35–1.52). These findings indicate increased copy numbers of exons 1–4 of the *MECP2* gene and its flanking regions in the chorionic villus sample, consistent with a duplication. Combined with the high‐throughput sequencing results, these findings supported a diagnosis of MDS in the fetus (Figure 3). Based on these results, the family pedigree is shown in Figure 4.

**FIGURE 3:**
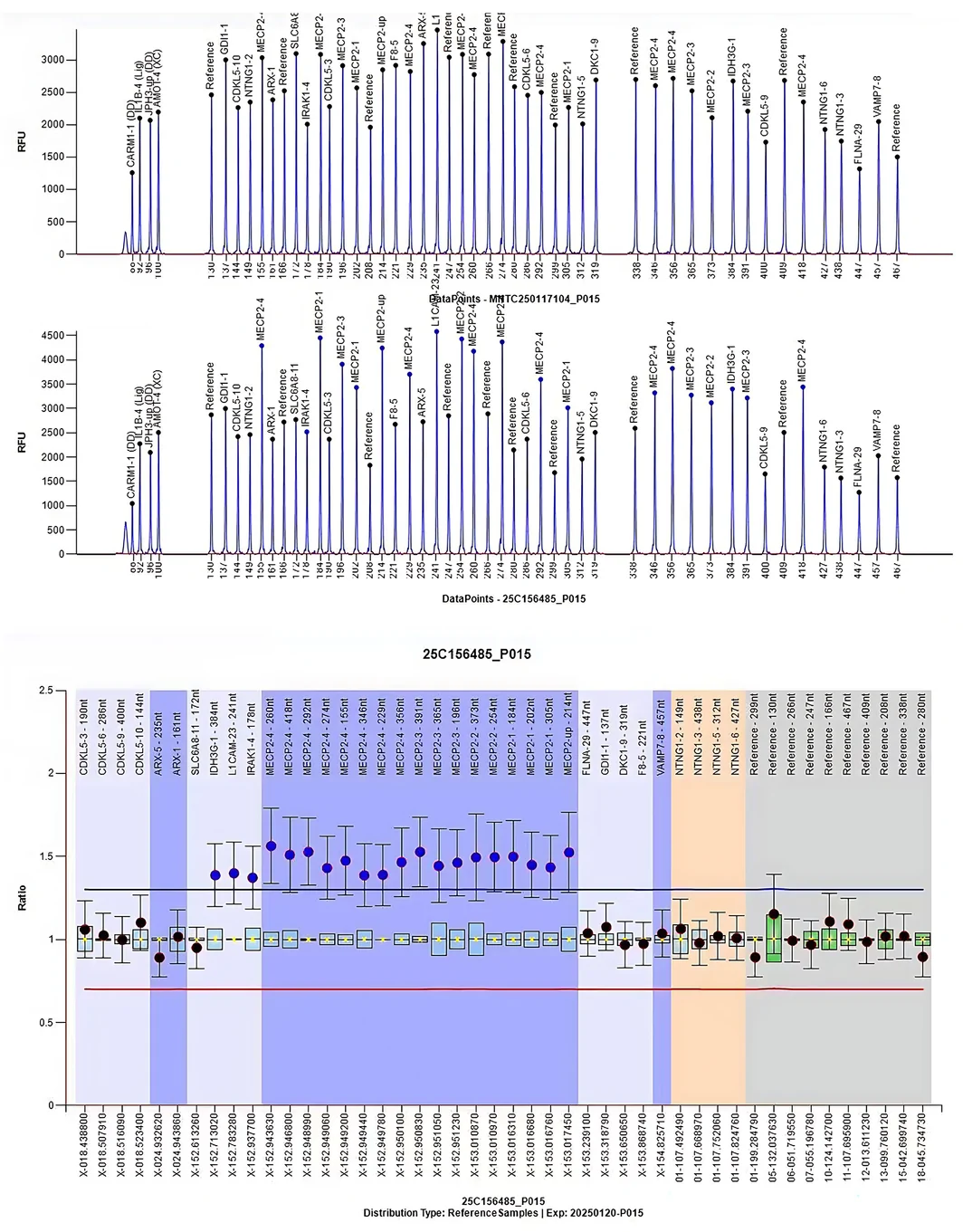
MLPA results. A fluorescence signal intensity between 0.7 and 1.3 was considered normal.

**FIGURE 4:**
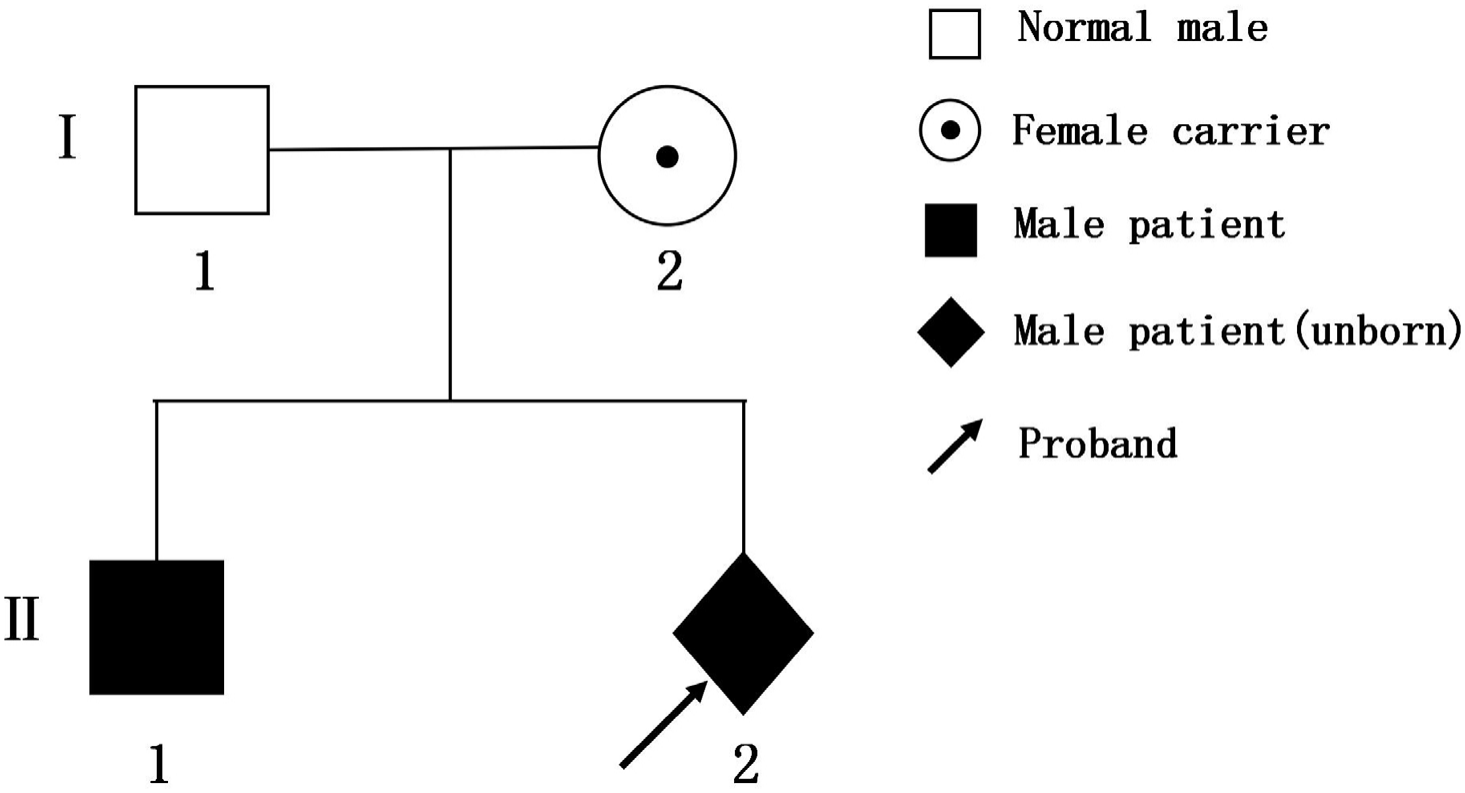
Pedigree of the family. I‐1 (proband's father): 46,XY (normal); I‐2 (proband's mother): 46,XX,dup(X) (q28) (asymptomatic carrier); II‐1 (proband's brother): 46,XY,dup(X)(q28) (affected); II‐2 (proband): 46,XY,dup(X)(q28) (affected).

### 3.3 Variant Interpretation

The Xq28 duplication involving *MECP2* was classified as pathogenic according to ACMG/ClinGen principles for CNV interpretation. Supporting evidence included its location within a known dosage‐sensitive region (ClinGen triplosensitivity score = 3), segregation with the affected male siblings in this family, absence of the duplication in the unaffected father, and the established association between *MECP2* duplication and MDS. The fetal *MECP2* duplication was independently confirmed by MLPA. Because the sequencing was performed at 0.2× depth, the precise CNV breakpoints were not considered definitively established.

## 4 Discussion

In this study, high‐throughput sequencing identified an Xq28 duplication in the fetus, the mother, and the proband's older brother. All reported duplicated segments involved the *MECP2* locus, which is the principal pathogenic gene responsible for MDS [14, 15]. *MECP2* dosage imbalance can disrupt neuronal development and synaptic function, and experimental studies have shown that *MECP2* overexpression is associated with abnormalities in synaptic plasticity and increased oxidative stress [16, 17, 18, 19]. The Xq28 region also contains repetitive sequences that may contribute to structural variation and differences in breakpoint mapping [20]. In the present family, the combination of CNV‐seq screening, MLPA confirmation, and pedigree analysis provided molecular evidence for prenatal diagnosis and maternal inheritance.

A key methodological limitation is the use of ultra‐low‐depth sequencing (0.2×) for a CNV of approximately 0.35–0.4 Mb. Published evidence indicates that some low‐coverage analytical methods can detect CNVs larger than 300 kb at approximately 0.2× depth [21], and low‐pass whole‐genome sequencing has also been clinically evaluated for CNVs > 50 kb [22]. However, comparative studies also show that ultra‐low‐coverage approaches can produce false positives for smaller CNVs and show variable performance on sex chromosomes [23]. Thus, the present CNV‐seq results should be interpreted as screening‐level evidence rather than definitive breakpoint characterization. The fetal *MECP2* duplication was independently confirmed by MLPA, which supports the presence of the pathogenic *MECP2* copy‐number gain.

The estimated duplication size differed slightly between the fetus (approximately 0.35 Mb) and the older brother (approximately 0.4 Mb). Because the sequencing depth was low and Xq28 contains repetitive and segmentally duplicated sequences, these coordinates and size estimates should be interpreted cautiously. The difference may reflect the resolution and boundary‐calling characteristics of low‐depth sequencing; low‐level mosaicism cannot be completely excluded, although no mosaicism assay was performed. Importantly, both CNV‐seq calls involved the *MECP2* locus, and MLPA independently confirmed increased copy numbers of *MECP2* exons 1–4 in the fetal sample, supporting the clinically relevant *MECP2* copy‐number gain.

The mother was clinically asymptomatic despite carrying the Xq28 duplication. Although skewed X‐chromosome inactivation has been reported in female carriers of *MECP2* duplications [24], no X‐inactivation assay was performed in this family. Therefore, we cannot determine whether skewed X‐chromosome inactivation explains the mother's asymptomatic phenotype. This remains an important limitation and should be addressed by X‐inactivation analysis in future studies. The reported immune abnormalities in some individuals with MDS [25] were not specifically evaluated in this family and therefore cannot be used to explain the mother's phenotype.

From a clinical perspective, this case illustrates the value of combining low‐pass CNV screening with a locus‐specific confirmatory assay for prenatal diagnosis and family testing. For families with a known *MECP2* duplication, molecular confirmation can facilitate genetic counseling and reproductive decision‐making. Clinical management remains largely supportive, including early rehabilitation and treatment of complications such as recurrent infections [26, 27], but these aspects are beyond the scope of the present case report.

## Author Contributions

FengQiu Lin and DingGuo Chen performed the research and drafted the manuscript; GuiDan Xu conceived the research; ZhengLin Lu and Yu Huang provided support for the experiments and manuscript writing, ChunFang Wang, JuHua Liang, and XueJuan Nong performed the research and collected the data; ZhengYi Chang and FuBo Wei analyzed the data. All authors reviewed and approved this manuscript.

## Funding

This study was supported by The Science and Technology Plan Project of Baise (grant/award number: BaiKe20211815; BaiKeZJ252818); The First Batch of Highlevel Talent Scientific Research Projects of the Affiliated Hospital of Youjiang Medical University for Nationalities (grant/award number:Y202011708); Open Competition Project of Guangxi Plateau Discipline (2026), Youjiang Medical University for Nationalities (grant/award number:YY2026GYY08).

## Ethics Statement

All procedures performed in studies involving human participants were in accordance with the ethical standards of the Institutional and/or National Research Committee and with the 1964 Helsinki Declaration and its later amendments or comparable ethical standards. This study was approved by the Medical Ethics Committee of Affiliated Hospital of Youjiang Medical University for Nationalities (approval no. YYFY‐LL‐2024‐299).

## Consent

For the part of the study involving human subjects, written informed consent was obtained from all participating patients and family members.

## Conflicts of Interest

The authors declare no conflicts of interest.

## Data Availability

The data that support the findings of this study are available from the corresponding author upon reasonable request.
